# Proteome Analysis Reveals a Significant Host-Specific Response in *Rhizobium leguminosarum* bv. viciae Endosymbiotic Cells

**DOI:** 10.1074/mcp.RA120.002276

**Published:** 2020-12-06

**Authors:** David Durán, Marta Albareda, Carlos García, Ana-Isabel Marina, Tomás Ruiz-Argüeso, Jose-Manuel Palacios

**Affiliations:** 1 Centro de Biotecnología y Genómica de Plantas (C.B.G.P.) UPM-INIA, Campus de Montegancedo, Universidad Politécnica de Madrid, Madrid, Spain; 2 Departamento de Biotecnología-Biología Vegetal, Escuela Técnica Superior de Ingeniería Agronómica, Alimentaria y de Biosistemas, Universidad Politécnica de Madrid, Madrid, Spain; 3 Servicio de Proteómica, Centro de Biología Molecular Severo Ochoa (CBMSO), CSIC Campus Cantoblanco, Madrid, Spain

**Keywords:** proteomics, bacteroids, NCR peptides, Pisum, lens, symbiosis, nitrogen fixation, legume, ABC, ATP-binding cassette, IRLC, intergenic region-lacking clade, iTRAQ, isobaric tags for relative and absolute quantitation, NCR peptide, nodule-specific cysteine-rich peptides, pI, isoelectric point, PSMs, peptide spectrum matches, PSP, periplasmic substrate–binding protein, sHSPs, small heat-shock proteins, TCA cycle, tricarboxylic acid cycle, USP, universal stress protein

## Abstract

The *Rhizobium*-legume symbiosis is a beneficial interaction in which the bacterium converts atmospheric nitrogen into ammonia and delivers it to the plant in exchange for carbon compounds. This symbiosis implies the adaptation of bacteria to live inside host plant cells. In this work, we apply RP-LC-MS/MS and isobaric tags as relative and absolute quantitation techniques to study the proteomic profile of endosymbiotic cells (bacteroids) induced by *Rhizobium leguminosarum* bv viciae strain UPM791 in legume nodules. Nitrogenase subunits, tricarboxylic acid cycle enzymes, and stress–response proteins are among the most abundant from over 1000 rhizobial proteins identified in pea (*Pisum sativum*) bacteroids. Comparative analysis of bacteroids induced in pea and in lentil (*Lens culinaris*) nodules revealed the existence of a significant host-specific differential response affecting dozens of bacterial proteins, including stress-related proteins, transcriptional regulators, and proteins involved in the carbon and nitrogen metabolisms. A mutant affected in one of these proteins, homologous to a GntR-like transcriptional regulator, showed a symbiotic performance significantly impaired in symbiosis with pea but not with lentil plants. Analysis of the proteomes of bacteroids isolated from both hosts also revealed the presence of different sets of plant-derived nodule-specific cysteine-rich peptides, indicating that the endosymbiotic bacteria find a host-specific cocktail of chemical stressors inside the nodule. By studying variations of the bacterial response to different plant cell environments, we will be able to identify specific limitations imposed by the host that might give us clues for the improvement of rhizobial performance.

## Highlights

• LC-MS/MS proteome of *Rhizobium* bacteroids in pea and lentil nodules.
• Legume host affects the presence of multiple rhizobial proteins in nodule bacteroids.
• A pea-specific GntR-like regulator contributes to symbiosis in pea nodules.
• Different profiles of plant-derived NCR peptides in pea and lentil bacteroids.

## Graphical Abstract

**Figure:**
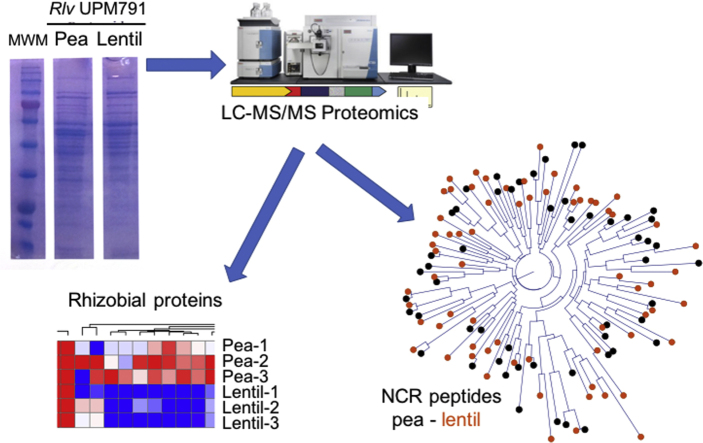

## In Brief

Comparison of proteomes from nodule bacteroids produced by a single *Rhizobium leguminosarum* strain in two different hosts (pea and lentil) reveals the existence of dozens of host-specific rhizobial proteins in the endosymbiotic cells, thus suggesting the requirement of rhizobia to adapt to different cellular environments. Host-specific sets of plant-derived nodule-specific cysteine-rich peptides were also found in the bacteroids.

The *Rhizobium*-legume symbiosis represents a model plant–microbe interaction in which both symbionts cooperate to convert atmospheric nitrogen into ammonia, thus allowing growth of nodulated plants in nitrogen-poor soils (1). The formation of nitrogen-fixing nodules in legume plants is the result of a controlled infection following a sophisticate exchange of chemicals between plant and rhizobia to recognize each other as compatible partners. Plant flavonoids and bacterial lipochitooligosaccharides (also designated as Nod factors) are the main signals involved in the recognition between the two symbiotic partners. Other structures, such as rhizobial exopolysaccharide and lipopolysaccharide, and plant lectins, also participate in this recognition (2). Rhizobial cells living inside legume nodules are modified into N_2_-fixing entities that feed the plant with ammonia, whereas the plant supports bacterial metabolism by supplying carbon substrates, essentially organic acids such as malate, and adequate micro-oxic conditions for nitrogenase expression (3). Many aspects of carbon, nitrogen, and oxygen metabolisms of the rhizobial vegetative cells are drastically changed to allow symbiotic nitrogen fixation in endosymbiotic cells (bacteroids) (1). Two main types or nodules, determinate and indeterminate, are produced by different types of legume plants. Indeterminate nodules are induced by the inverted repeat-lacking clade (IRLC) group of legume plants, which includes relevant crops such as *Medicago* and *Pisum*. This type of mainly elongated nodules keep meristematic activity along nodule lifespan, with bacteroids that undergo a terminal differentiation process with profound ultrastructural and morphological modifications; non-IRLC plants (like *Glycine* and *Phaseolus*) induce determinate nodules that are round, with no terminal differentiation of the bacteroids (4).

The degree of specificity of the *Rhizobium*-legume interaction is variable depending on the systems, and a wide range of possibilities from extremely specific symbiosis (only 1 legume cultivar and 1 specific strain) to highly promiscuous (a single strain able to nodulate over 300 legume species) have been described (5). *Rhizobium leguminosarum* bv viciae (*Rlv*) participates in symbioses with an intermediate degree of specificity. Although *Pisum sativum* is considered as the “standard” host for this rhizobial species, *Rlv* also effectively nodulates legumes from other IRLC genera within the Vicieae tribe such as *Lathyrus*, *Vicia*, and *Lens* (5). However, the shared ability of forming effective, nitrogen-fixing nodules with the same rhizobial strain does not mean that all these hosts originate identical symbioses. In fact, host-specific differences on the expression of rhizobial traits relevant for nitrogen fixation have been described. Previous work carried out in our laboratory had shown a marked effect of the host on the expression of hydrogenase, a rhizobial metalloenzyme that recycles hydrogen evolved from nitrogenase during the nitrogen-fixation process. This enzyme is induced in bacteroids from *Pisum*, *Vicia*, and *Lathyrus*, but not from *Lens* (6). The molecular mechanism(s) responsible for the host effect on hydrogenase expression has not been fully elucidated yet, although it has been demonstrated that it is exerted at both transcriptional and posttranscriptional levels (6). A study of *Bradyrhizobium japonicum* symbioses with soybean, siratro, and cowpea revealed the existence of host-specific proteomic and metabolomic bacteroid profiles (7, 8). In contrast, a transcriptome-based analysis carried out in *R. leguminosarum* bv viciae 3841 in symbiosis with pea and vetch revealed pea-specific ribosomal RNA processing, but no significant host-dependent differences in the level of expression of the genes upregulated in bacteroids (9). The effect of the host in symbiotic associations sharing the same rhizobial strain might reflect differences in the intracellular environment of the microsymbiont. Host-derived compounds affecting the microsymbiont have been described in legume plants from the IRLC taxon. These legumes produce a large battery of nodule-specific cysteine-rich (NCR) peptides structurally similar to antimicrobial defensins (10, 11). NCRs are sent to the bacteroids through a specialized secretory pathway that is essential for nitrogen fixation in *Medicago truncatula* nodules (12). It has been shown that these peptides are able to induce modifications in rhizobial vegetative cells similar to those present in bacteroids (13). The number and type of symbiotic NCR peptides correlate with bacteroid morphotypes and vary according to the legume species, ranking from just a few to over 600 different peptides (14). It is assumed that these peptides are main actors controlling bacterial physiology in indeterminate legume nodules (15). The comparative study of symbiotic systems involving two related legume hosts nodulated by the same rhizobial strain might shed light on the relevance of these peptides on symbiotic performance.

Global analyses in cellular systems have been greatly facilitated by the development of novel MS-based methods allowing extensive proteomic characterization of complex samples such as legume nodules (16). Although a detailed proteomic atlas is available for the model legume *M. truncatula* (17), no report of proteomic analysis of *R. leguminosarum* bv viciae bacteroids has been published to date. Transcriptomic analysis, although powerful by the ability of giving sequence information for every single gene in the organism, has some limitations because posttranscriptional regulation is revealing as more and more complex, and situations of weak correlation between RNA and protein abundance have been reported (18). The proteome represents a closer proxy to the phenotype, on which selection acts, than the transcriptome (19). In this work, we use LC-MS/MS–based proteomics to characterize bacteroids induced by *Rlv* UPM791 in nodules of two different legume plants (pea and lentil). The data indicate the existence of a significant host-specific differential response affecting a number of proteins likely involved in the adaptation to specific host conditions. Significant differences were also found on the set of NCR peptides sent by each host plant to the bacteroids.

## Experimental Procedures

### Biological Material

Pea (*P. sativum* cv. Frisson) and lentil (*Lens culinaris* cv. Magda) nodules were obtained from plants inoculated with *Rlv* UPM791 (20) and grown under bacteriologically controlled conditions as described (6). Nodules from 21-days (pea) or 28-day-old (lentil) plants were harvested, subsequently frozen in liquid nitrogen, and stored at −80 °C until further use.

### RNA Extraction

For total RNA extraction, nodule samples were ground in a cold mortar with 500 μl of TRIzol reagent (Sigma-Aldrich) and transferred into a microfuge tube. Following incubation for 5 min at RT, 100 μl of chloroform was added, and after mixing, samples were incubated for 3 min at RT and centrifuged (12,000*g*, 15 min); RNA was precipitated overnight at −20 °C in the presence of 1 μl of glycogen and 250 μl of isopropanol. Following centrifugation (15,000*g*, 10 min at 4 °C), the pellet was washed with 1 ml of 75% ethanol and centrifuged (15,000*g*, 1 min) and resuspended in RNAse free water (Sigma-Aldrich), incubated at 60 °C for 5 min, and transferred to ice. Then, 5 μl of DNAse buffer, 2.5 μl of Turbo DNAse (Invitrogen, ThermoFisher), and 2.5 μl of RNAse-Out (Fisher Scientific) were added to the sample and incubated for 30 min at 37 °C. RNA samples were then purified with NucleoSpin-RNA kit (Macherey-Nagel) following manufacturer’s specifications. RNA concentration was quantified with a Nanodrop spectrophotometer and a Qubit 2.0 Fluorometer (Life Technologies). The quality of the RNA samples was checked using an Agilent RNA 6000 Nano kit and 1% electrophoretic agarose gel in a Bioanalizer assay (Agilent). The absence of DNA in the samples was checked with control PCR reactions: in the case of UPM791 strain, primers rpoD-F (5ʹ-ACGACTGACCCGGTACGCATGTA-3ʹ) and rpoD-R (5ʹ-ATAGAAATAACCAGACGTAACTT-3ʹ) were used; PLC-16/PLC-22 (21) and AB-72/AC-58 (22) primer pairs were used to detect the presence of residual DNA in lentil and pea, respectively.

### Protein Extraction

Protein samples were obtained from *Rlv* UPM791 pea and lentil bacteroids as previously described (23) and collected in 500 μl of 100 mM Tris-HCl buffer (pH 7.2). For vegetative cells, 10 ml of TY medium (6) was inoculated with *Rlv* UPM791 strain and grown at 28 °C shaking (200 rpm) until an optical density of 0.6 was reached. Bacterial samples were concentrated by centrifugation (8000*g*, 10 min), washed three times with Tris-HCl buffer, and resuspended in 500 μl of 100 mM Tris-HCl buffer (pH 7.2). Bacterial cells were disrupted on ice with cyclic sonications (15” sonication and 20” pause, 15 cycles on a Branson 150 sonifier). Cellular debris and insoluble materials were removed by centrifugation at 16,000*g* for 10 min. Sample quality and concentration were checked by Qubit and gel profile. Finally, protein extract was lyophilized and kept at −80 °C until used.

### RNA Sequencing and Assembly

RNA samples were processed at the National Center of Genomic Analysis—Center for Genomic Regulation. Processing included a poly(A) enrichment and construction of libraries using the Illumina’s TruSeq Stranded mRNA Library Prep Kit (Life Technologies). Libraries were paired-end sequenced (>65 M of reads, Read length 2 × 100 bp) as recommended by Illumina (https://www.illumina.com), using an Illumina HiSeq machine in high output mode with one lane per library. *De novo* assembly of RNA-Seq data was performed using Trinity platform (https://galaxy.ncgas-trinity.indiana.edu/; [24]), and the assembly obtained was visualized and analyzed using the Geneious Pro software (version 5.6.5; Biomatters).

### Protein Extract Digestion and Proteomic Analysis

Protein extracts were subjected to in-solution tryptic digestion and desalted as previously described (25). The desalted protein digests were dried, resuspended in 10 μl of 0.1% formic acid, 4% acetonitrile and analyzed by RP-LC-MS/MS in an Easy-nLC II system coupled to an ion trap LTQ-Orbitrap-Velos-Pro hybrid mass spectrometer (Thermo Scientific). Peptide identification from raw data was carried out using the SEQUEST algorithm (Proteome Discoverer 1.4, Thermo Scientific). Database search was performed against *Rlv* UPM791 genome-derived proteome (7480 entries, 26), Uniprot-Fabaceae.fasta (356,432 entries), and *de novo* assembly of RNA-Seq data from pea and lentil nodules (284 and 394 entries, respectively). The following constraints were used for the searches: tryptic cleavage after Arg and Lys, up to two missed cleavage sites, and tolerances of 20 ppm for precursor ions and 0.8 Da for MS/MS fragment ions; searches were performed allowing optional Met oxidation and Cys carbamidomethylation. A search against a decoy database (integrated decoy approach) was carried out using false discovery rate < 0.01. Detailed conditions for the LC-MS/MS analysis are provided as Supplementary Material (25, 26, 27, 28). Estimation of protein abundance was carried out through comparison of RPSM values, calculated as RPSM=(PSM/number of predicted tryptic peptides) × 100. The number of predicted tryptic peptides for each protein was calculated using Protein Digestion Simulator software (PNNL, available from https://omics.pnl.gov/software/protein-digestion-simulator).

To search for NCR peptides not present in the databases used, we generated a *de novo* list including all MS/MS spectra that did not correspond to any compiled database peptides using PEAKS software (Bioinformatics Solutions Inc). This *de novo* list was used to identify NCR peptides. First, we generated an alignment of all the NCR sequences present in UniProt DB and look for all the conserved sequences. We manually searched in the *de novo* MS/MS spectra with tags of three amino acids. Candidates were used for the identification of NCR peptides based on homologies using BLAST in databases and in the *in silico* translated proteins from the libraries of pea and lentil nodule RNA-seq derived contigs.

### iTRAQ Labeling and Analysis

The protein extracts tryptic digest (100 μg) were labeled using chemicals from the iTRAQ reagent 8plex Multi-plex kit (Applied Biosystems) essentially as described (26). Briefly, peptides were dissolved in 0.5 M triethylammonium bicarbonate, adjusted to pH 8. For labeling, each iTRAQ reagent was dissolved in 50 μl of isopropanol and added to the respective peptide mixture and then incubated at RT for 2 h. Labeling was stopped by the addition of 0.1% formic acid. Whole supernatants were dried down, and the samples were mixed to obtain the labeled mixture. The mixture was desalted onto OASIS HLB Extraction Cartridges (Waters Corporation) and kept at −80 °C until the mass spectrometric analysis. Processing sites of NCR peptides identified were *in-silico* predicted using SignalP 3.0 server (http://www.cbs.dtu.dk/services/SignalP-3.0). Isoelectric points of processed peptides were determined using the SMS software package (29) at http://groups.molbiosci.northwestern.edu/matouschek/links/sms2/protein_iep.html.

### Construction of RLV_1934A Mutant

To generate a mutant in *gntR* gene (RLV_1934A), a pK18*mob* plasmid was inserted into the *Rl*v UPM791 wild-type copy of the gene by a single crossover event. For doing this, an internal fragment of 272 bp was amplified with primers, 292_GntR_int_F (5ʹ-ACGAGACCGATGTCGGAAAG-3ʹ) and 293_GntR_int_R (5ʹ-CCGAAGTTCCGACGCAGTTA-3ʹ), and cloned into pCRTM2.1-TOPO TA CloningKit (Invitrogen, Life Technologies). The fragment was sequenced, cloned as an *Eco*RI-*Bam*HI fragment in pK18*mob* suicide vector (30), and the resulting plasmid was conjugated into *Rlv* UPM791. Single-crossover positive colonies were selected by plating on Rmin medium (6) supplemented with kanamycin. Insertion was verified by PCR analysis using appropriate primers, and a UPM791*gntR*::pK18*mob* clone was selected and designated UPM1418.

For complementation studies, plasmid pBBGntR was constructed. For this purpose, DNA containing 205 pb upstream and the whole protein-encoding region of RLV_1934A gene was PCR-amplified using primers 315_GntR_F_ext (5ʹ-AATTAGTGGCAGAATGCGAT-3ʹ) and 316_GntR_R_ext (5ʹ-TCACAAACTTCTCGCAGGCC-3ʹ). The resulting DNA fragments were cloned into pCR 2.1TOPO vector; the correct sequence of the constructions was confirmed by sequencing, and then, the region was cloned into the broad host range vector pBBR1-MCS5 (31) as a *Kpn*I-*Apa*I frament. The resulting plasmid, pBBGntR, was mated into *gntR* mutant UPM1418, thus generating UPM1419 strain.

### Plant Tests

For the plant inoculation test, experiments were carried out in sterile Leonard jars containing N-free nutrient solution (32) with vermiculite as substrate. Pea and lentil surface-sterilized seeds (ethanol 70% and bleach 12.5%) were germinated on 1% agar plates, and seedlings were inoculated with 1 ml of early stationary phase bacterial cultures. Plants were grown under bacteriologically controlled conditions in greenhouse using 16/8 h day/night light cycles at 25/23 °C; 21 days postinoculation, pea (28 days for lentil) plants were harvested, and shoots were dried in an oven at 60 °C for 48 h. Total nitrogen content of the shoot was determined using a TruMac C/N analyzer (Leco Corporation).

### Experimental Design and Statistical Analysis

Three independent replicates of pea and lentil bacteroids were used for RPSM determinations, and two replicates were used for vegetative cells and for iTRAQ determinations. RPSM and iTRAQ data are included in supplemental Table S1, and raw data are available at PRIDE repository (33).

Statistical analysis of plant assays was performed by ANOVA linear model test, following a completely random design. Multiple comparisons of means were analyzed by Fisher’s protected least significant difference method. The analysis was performed using Statistix 10 software.

## Results and Discussion

### Proteome Analysis of *R. leguminosarum* bv viciae Pea Bacteroids

Protein extracts from bacteroids induced by *Rlv* UPM791 in pea nodules were digested with trypsin and analyzed through LC-MS/MS, and the resulting set of spectra were matched with the *in silico* digested proteome deduced from *Rlv* UPM791 genome. In this analysis, we also included extracts from TY-grown vegetative cells of the same strain as a reference list. A total of 1104 rhizobial proteins were detected in pea bacteroid extracts, whereas 1324 proteins were detected in the case of vegetative cells (Table 1 and supplemental Table S1). The distribution of the corresponding genes among the six replicons of the strain (chromosome and five plasmids, Table 1) revealed that over 80% of proteins detected in pea bacteroids were encoded in the chromosome, well over the share of this replicon on the total genome (61%). Four out of the five plasmids present in *Rlv* UPM791 contributed a percentage of proteins substantially lower than their share relative to the genome in bacteroids (Table 1). The exception was pUPM791c, which contributed to the bacteroid proteome as many proteins as expected from its size. pUPM791c is the symbiotic plasmid of the strain, encoding the *nod*, *nif*, and *fix* genes known to participate in the symbiosis. In the case of vegetative cells, the percentage of proteins coming from the extrachromosomal DNA was much lower than its genome share, confirming the low percentage of genes from these replicons expressed under culture conditions. These results are in line with the concept of secondary replicons as reservoirs for niche adaptation (34).

**Table 1:** Distribution of proteins^a^ detected in pea and lentil bacteroids in the *Rlv* UPM791 replicons

Replicon	Total proteins encoded^b^	Vegetative cells^c^	Pea bacteroids		Lentil bacteroids	
			Proteins identified^c^	Host specific^d^	Proteins identified^c^	Host specific^d^
Chromosome	4587 (62.7%)	1154 (87.1%)	901 (81.6%)	12	891 (85.3%)	13
pRlvA	1246 (17.0%)	62 (4.7%)	50 (4.5%)	4	42 (4.0%)	8
pRlvB	588 (8.0%)	44 (3.3%)	50 (4.5%)	3	35 (3.3%)	2
pRlvC	366 (5.0%)	10 (0.7%)	68 (6.2%)	7	50 (4.8%)	1
pRlvD	239 (3.3%)	8 (0.6%)	3 (0.3%)	0	4 (0.4%)	-
pRlvE	545 (7.4%)	46 (3.5%)	32 (2.9%)	2	22 (2.1%)	1
Total	7318	1324	1104	28	1044	25

Numbers in brackets represent the percentage of proteins in each replicon referred to the total number of proteins on each column.

a Proteins detected with at least two tryptic peptides.

b According to Sanchez-Canizares *et al.* (35).

c Total proteins identified in one or more replicates.

d Proteins present in the three replicates from one host (with ten or more accumulated spectra) and absent in all replicates from the other host.

Proteins identified in pea bacteroid extracts were analyzed for subcellular localization using PSORTb V.3.0 algorithm (35). Only 11% proteins whose localization was predicted (99 out of 929) were considered as membrane proteins (supplemental Table S1). A significant fraction of membrane proteins in a cell are components of transport systems. Within these, ATP-binding cassette (ABC)-type transporters constitute an ubiquitous group of ATP-powered transport systems that include one to two nucleotide-binding proteins and also transmembrane protein(s) that mediate substrate transport across the cytoplasmic membrane. Most uptake ABC transporters rely on a periplasmic component (from periplasmic substrate–binding protein [PBP]) that bind specific substrates with high affinity, thus conducting it to the transport components in the membrane (36). The genome of *Rlv* UPM791 encodes 183 such ABC transport systems (35), from which 54 PBPs were identified in the pea bacteroid extract (supplemental Table S1). However, only in one case, the cognate membrane protein (amino acid transporter RLV_4521) was also identified in the proteome (supplemental Table S1). Because these membrane proteins are usually part of the same operon as the PBPs, this preferential detection of the periplasmic component likely indicates the limited ability of our system to detect integral membrane proteins. The low number of membrane proteins detected (as compared with 33% membrane-associated proteins predicted in the genome) is probably because of the extraction procedure, which included ultracentrifugation steps not designed for the extraction of particulate fractions. Thus, a lower efficiency in the detection of membrane-associated proteins is expected.

The number of peptide spectrum matches (PSMs) detected for each protein represents the number of times that peptides derived from a given protein are detected. This parameter has been used to estimate the abundance of a protein in the extract (7, 16, 37). We used a derived parameter based on a normalization of the PSM (RPSM, normalized with reference to the number of potential tryptic peptides in the corresponding protein, see Experimental Procedures section) as an approximate estimation of the abundancy of the protein. Mean values for RPSM for proteins found in all replicates are included in supplemental Table S1. The data indicate a reasonable reproducibility among biological replicates (dispersion lower than 30% for PSM means >20, supplemental Fig. S1), taking into account the heterogenous nature of the nodule material. Based on RPSM parameter, we generated a short list including the 50 proteins with highest RPSM values (ranging from 93 to 380) and likely highly abundant in pea bacteroids (Table 2). Twenty-eight out of the 50 proteins corresponded to three main functional groups: nitrogenase-related proteins, enzymes of the central carbon/nitrogen metabolisms, and chaperone/stress–response proteins.

**Table 2:** *Rlv* UPM791 proteins showing the highest relative amounts of detected spectra in proteomic analysis of pea bacteroid extracts

Accession	Description	Protein size (aa)	RPSM^a^	
			Pea bacteroids	Vegetative cells
RLV_1841	nitrogenase_molybdenum-iron_protein_beta_chain_NifK	514	379.3	n.d.
RLV_6653	ATP_synthase_subunit_beta	479	296.8	183.9
RLV_6687	malate_dehydrogenase	321	286.7	135.0
RLV_3265	molecular_chaperone_GroEL	547	284.8	278.3
RLV_1842	nitrogenase_molybdenum-iron_protein_alpha_chain_NifD	495	264.2	n.d.
RLV_1843	putative_nitrogenase_iron_protein_NifH	298	200.0	n.d.
RLV_3292	photosystem_reaction_center_subunit_H	202	193.9	22.7
RLV_7110	4-aminobutyrate_aminotransferase	427	192.2	40.0
RLV_1844	diaminobutyrate_aminotransferase	425	182.2	n.d.
RLV_4155	histone-like DNA binding protein HU	92	181.5	822.2
RLV_6681	dihydrolipoamide_succinyltransferase	422	177.4	103.2
RLV_6296	heat-shock_protein_IbpA	157	169.4	n.d.
RLV_1384	universal_stress_protein	278	166.7	n.d.
RLV_5727	membrane_fusogenic_activity	87	156.7	95.0
RLV_1399	molecular_chaperone_Hsp20	170	145.8	n.d.
RLV_6684	succinyl-CoA_synthetase_subunit_alpha	301	143.3	102.5
RLV_4551	type_II_citrate_synthase	430	142.4	68.2
RLV_7164	molecular_chaperone_DnaK	639	141.7	132.7
RLV_4578	membrane_protein	128	139.4	n.d.
RLV_1979	isocitrate_dehydrogenase	404	137.3	60.7
RLV_4899	isocitrate_dehydrogenase	404	135.8	140.2
RLV_4236	elongation_factor_Tu	392	135.2	232.9
RLV_1843A	cytochrome_C_biogenesis_protein	153	130.8	n.d.
RLV_7109	NAD-dependent_succinate-semialdehyde_dehydrogenase	494	129.5	n.d.
RLV_4347	universal_stress_protein	281	129.2	n.d.
RLV_1895B	NifT/FixU_protein	69	128.6	n.d.
RLV_1848	Glutamyl-tRNA_reductase	316	128.0	n.d.
RLV_3309	ATP_synthase_subunit_b_1	164	126.2	146.4
RLV_4004	acyl_carrier_protein	79	125.0	-
RLV_4846	ABC_transporter_ATP-binding_protein	252	124.2	59.1
RLV_7032	thioredoxin	107	123.8	185.7
RLV_969	putative_60_kDa_chaperonin	543	123.2	109.8
RLV_3266	molecular_chaperone_GroES	99	120.5	207.7
RLV_6777	aconitate_hydratase	897	120.4	43.3
RLV_4395	peroxidase	220	117.8	53.3
RLV_6223	hypothetical_protein	63	116.7	141.7
RLV_6211	glyceraldehyde-3-phosphate_dehydrogenase	337	116.0	100.0
RLV_1884	lysine_2,3-aminomutase	375	113.2	n.d.
RLV_1821	OsmC-like_protein	184	111.1	n.d.
RLV_1846	D-alanine--D-alanine_ligase	359	110.7	n.d.
RLV_6655	ATP_synthase_subunit_alpha	510	110.6	103.4
RLV_1394	peptidoglycan-binding_protein	216	107.6	n.d.
RLV_6215	fructose-bisphosphate_aldolase	342	106.3	90.6
RLV_6686	succinyl-CoA_ligase_subunit_beta	398	105.4	86.5
RLV_252	catalase-peroxidase	729	105.1	68.6
RLV_2993	translation_initiation_factor_IF-1	73	100.0	70.0
RLV_5214	cold-shock_protein	71	100.0	285.7
RLV_6683	2-oxoglutarate_dehydrogenase_subunit_E1	1019	98.1	61.0
RLV_4730	universal_stress_protein_UspA	274	95.8	n.d.
RLV_4084	6,7-dimethyl-8-ribityllumazine_synthase	152	93.3	-

n.d., not detected; -, present in only 1 replicate.

a RPSM represents the number of peptide spectra matches (PSMs) normalized to the number of tryptic peptides predicted for the protein (see text for details).

#### Nitrogenase-Related Proteins

Nitrogenase structural subunits NifKDH (RLV_1841–1843) were among the most abundant proteins in pea bacteroids, thus confirming the specialization of bacteroids in the conversion of atmospheric nitrogen into ammonia. These data are consistent with previous transcriptomic analysis in pea bacteroids from *Rlv* 3841 (9) and also in bacteroids from other symbiotic systems (16). In addition to nitrogenase structural proteins, a NifT/FixU-like protein was also found in this group of prominent proteins. *Rlv* UPM791 NifT/FixU is a 68-aa long protein (RLV_1895B) encoded in the symbiotic plasmid 270 bp downstream from the *nifAnifBfer1* operon. This protein is conserved in different rhizobia, but its function has not been determined so far. A NifT/FixU-like protein is not annotated in the *Rlv* 3841 genome (38), although a CDS encoding a highly similar protein is present at the same relative position in the symbiotic plasmid of this strain (pRL10). The high level of NifT protein detected in pea bacteroids and its physical linkage to other genes involved in nitrogenase function in the *fixABCXnifABfer1nifT* region in several rhizobia strongly suggest the participation of this protein in nitrogenase synthesis or function. The remaining Nif and Fix proteins required for synthesis and functioning of nitrogenase and whose genes are organized in four operons in the *Rlv* UPM791 symbiotic plasmid (NifEN, NifAB, FixABCX, and FixNOQPGHIS; [35]) were all detected in our analysis, although at lower level, with the exception of FixQ and FixS, which were not detected (supplemental Table S1). The small size of these two proteins (50–52 residues) and their membrane-bound character (39, 40) likely contribute to their lack of detection in the present study.

#### Proteins Involved in Central Carbon and Nitrogen Metabolisms

The short list of highly abundant proteins in pea bacteroid extracts includes most enzymes of the tricarboxylic acid cycle (TCA) pathway: malate dehydrogenase, citrate synthase, aconitate hydratase, isocitrate dehydrogenase, oxoglutarate dehydrogenase, and succinyl-CoA synthetase (Table 2). These enzymes catalyze the different steps for the conversion of malate to succinate within the cycle. The other two components of the canonical TCA cycle (succinate dehydrogenase and fumarate hydratase) were also detected, although with a lower RPSM value (supplemental Table S1). The abundant presence of TCA enzymes in bacteroids is consistent with a malate-based carbon metabolism shown by previous transcriptomic and metabolomic analyses (9, 41) and generally accepted for the endosymbiotic state of pea bacteroids (3). Similarly, high levels of expression for TCA enzymes were previously identified in *B. japonicum* (16); more recently, citrate synthase was identified as a highly expressed gene in *Bradyrhizobium* sp. bacteroids induced in *Aeschynomene indica* (42). The genome of *Rlv* UPM791 (35) carries two copies of the gene encoding isocitrate dehydrogenase: a chromosomal copy (RLV_4899) and a pSym-located copy that is induced under microaerobic conditions (RLV_1979). Both copies show a high level of expression in pea bacteroids, although the high-sequence identity leaves a single specific peptide to discriminate between both isoforms.

The bacteroid malate-based metabolism prevalent under symbiotic conditions implies synthesis of 5-C and 6-C compounds through gluconeogenesis, usually starting from pyruvate (43). Enzymes involved in two routes for pyruvate synthesis described in rhizobia (NAD-dependent malic enzyme and the combination of phosphoenolpyruvate carboxykinase and pyruvate kinase; [44]) were consistently detected in pea bacteroids (RLV_4936, RLV_7044, and RLV_6265, respectively, in supplemental Table S1). In addition, three enzymes involved in different gluconeogenesis steps (fructose bisphosphate aldolase, RLV_6215; glyceraldehyde 3P dehydrogenase, RLV_6211; and enolase, RLV_4557; supplemental Table S1) were present in the pea bacteroid proteome, indicating an active gluconeogenic activity under symbiotic conditions.

The two enzymes of the glyoxylate shunt (isocitrate lyase and malate synthase) are encoded in the *Rlv* UPM791 genome (RLV_3156 and RLV_7060, respectively, (35)), but these proteins were not detected in the proteomic profile of pea bacteroids. This is consistent with the fact that malate synthase is not essential for nitrogen fixation in *R. leguminosarum* (45). In contrast, transcriptomic analysis of *Rlv* 3841 strain revealed a strong induction of malate synthase in pea bacteroids without concomitant induction of isocitrate lyase (9). We do not have a clear explanation for this discrepancy, that could be because of posttranscriptional regulation effects or to strain-specific differences.

The short list of prominent pea bacteroid proteins also include homologues to two enzymes involved in C/N metabolism, namely gamma-aminobutyrate aminotransferase (GABA-AT, RLV_7110) and succinate semialdehyde dehydrogenase (RLV_7109). These two enzymes have been described in the process of incorporation of GABA from the plant to yield glutamate and succinate (46). Interestingly, a protein (RLV_1844) showing a high similarity to a diaminobutyrate aminotransferase and encoded in the symbiotic plasmid was also highly abundant in pea bacteroids. This enzyme might participate either in the utilization of homoserine or in the degradation of ectoine. Both functions could be occurring in the pea nodule (47). Alternatively, this enzyme might be involved in the degradation of GABA, as it has been described that GABA can be also recognized by diaminobutyrate aminotransferase or participate in the synthesis of other amino acids. Phenotypic and metabolomic analyses of specific mutants, currently underway in our laboratory (Ballesteros *et al*., unpublished), are required to elucidate the actual role of this enzyme.

#### Stress-Related Proteins

A high number of stress–response proteins, including seven small heat-shock proteins (sHSPs) and ten universal stress proteins (USPs) are encoded in the genome of *Rlv* UPM791 (35), suggesting the presence of complex stress-responsive circuits in this bacterium.

The short list of abundant proteins in pea bacteroids includes two sHSPs (RLV_1399 and RLV_6296). The *Rlv* 3841 orthologs for these two proteins (RL1883 and RL4089, respectively) were described as highly induced in the transcriptomic comparison of mature pea bacteroids *versus* vegetative cells (9). Also, Smb21295, the *Sinorhizobium meliloti* homolog to RLV_6296, was found as bacteroid-specific in *Medicago* nodules (48). sHSPs are chaperones able to bind unfolded proteins, keeping them in a soluble, folding-competent state so they can be refolded with the assistance of ATP-dependent chaperones (49). Most bacteria contain one to two of such proteins, but some groups, and notably rhizobia, contain multiple members of this chaperone family. The potential role of these proteins as stress protectants in legume endosymbiotic bacteria has not been demonstrated so far. The presence of several heat-regulated sHSPs has been documented in *Bradyrhizobium* and *Mesorhizobium* strains (50), but no connection of the expression of these proteins with the symbiosis had been established. Previous functional evidence indicates the involvement of sHSPs in resistance to abiotic stress (desiccation) in *Azotobacter vinelandii* cysts (51).

The top protein list of pea bacteroid proteome contains other stress–response proteins: two USPs (RLV_4730 and RLV_1384), one cold-shock protein (RLV_5214), and also a OsmC-like protein (RLV_1821), along with subunits of general chaperones GroESL and DnaK and a 60-kDa chaperonin (RLV_969). Other stress-related proteins were present in the pea bacteroid proteome at lower relative abundance (supplemental Table S1). Interestingly, none of the sHSPs, USPs, or OsmC-like proteins highly expressed in bacteroids were detected in the proteome of vegetative cells (Table 1). Multiple stress-related chaperones and HSPs were also found in previous proteomic studies in *S. meliloti* bacteroids induced in *M. truncatula* nodules (48). Such a complex profile of stress-responsive proteins suggests that legume bacteroids are subjected to significant, symbiosis-specific stress within the nodule. The intracellular state in different eukaryote/prokaryote symbiotic associations is considered as a stressful condition, and it has been proposed that the stress response contributes to the stability of the symbiotic system (52). In the case of legume nodules, the microsymbiont is affected by physical stressors, such as the ultra-low oxygen tension to protect nitrogenase, and also by the production of reactive oxygen species by the plant and the bacterium (53). In the case of IRLC legumes such as pea, the presence of NCR-type antimicrobial peptides is an additional factor of stress. In fact, this type of peptides has been found in this proteomic analysis (see below).

#### Other Prominent Proteins

The list of proteins abundant in pea bacteroids includes a glutamyl-tRNA reductase (RLV_1848), encoded in the symbiotic plasmid and conserved in many rhizobium strains. In most bacteria, this protein participates in the C_5_ pathway for the synthesis of delta-aminolevulinic acid, a precursor of tetrapyrrol present in heme groups (54). The relevant amount of this protein is likely linked to the higher requirements of heme in bacteroids (54).

We also identified a protein (RLV_5727) belonging to the *Brucella* membrane fusogenic protein superfamily (55). The *Brucella* ortholog (Mfp, 71% identical) is required for full persistence of the pathogen in mice, with a proposed role in the fusion between *Brucella*-containing vesicles and the endoplasmic reticulum (56). In addition, it has been recently demonstrated that the function of the *Escherichia coli* member of this family is related to the synthesis of ubiquinone (57, 58).

Finally, several other proteins involved in general cell functions were also present in the list of highly expressed proteins, namely a DNA-binding histone-like protein (the protein giving the highest RPSM score in vegetative cells) and components of ATP synthase (Table 2), among others. Interestingly, only one ribosomal component (elongation factor Tu RLV_4236) was identified in that list of highly expressed bacteroid proteins. In contrast, ribosomal proteins constitute the largest group within the highly expressed in vegetative cells (22 out of 50, supplemental Table S1) in line with data from comprehensive quantification of *E. coli* proteins (59). These data suggest a lower level of protein synthesis in the endosymbiotic form of *Rhizobium* cells which is consistent with the inhibition of cell division described for these cells (13).

### Differential Protein Profiles in Pea *Versus* Lentil Bacteroids

*Rlv* UPM791 was originally isolated from a *P. sativum* root nodule (20). However, this rhizobial species is able to effectively nodulate legume plants from other genera, namely *Vicia*, *Lathyrus*, and *Lens*, and each plant species might provide a different cellular environment to bacteroids. It is known that the expression of at least one bacterial enzyme relevant for the symbiosis (NiFe hydrogenase) is host-dependent (6). To study whether host-specific environments might affect the expression of other rhizobial proteins, the proteomic profile of bacteroids induced by *Rlv* UPM791 in lentil nodules was determined. Using a procedure similar to that described above for pea bacteroids, a total of 1044 rhizobial proteins were detected in lentil bacteroids (supplemental Table S1 and Supplemental Fig. S2), and this list was compared with proteins identified in pea bacteroids. To minimize the possibility of false positives, a stringent selection of proteins was made by choosing only those identified in all three replicates of bacteroids from one host and absent in all replicates from the other host. Furthermore, because proteins showing a low number of spectra, close to the detection limit, also showed lower reproducibility among replicates (supplemental Table S1), we considered only proteins with an accumulated value of 10 or more spectra detected. Selection of host-dependent bacterial proteins under these conditions led to the identification of 28 proteins specifically detected in pea and 25 lentil-specific proteins (Table 3). As a complementary approach, comparison of bacteroid extracts was also carried out through quantitative isobaric tags for relative and absolute quantitation (iTRAQ) analysis of bacteroid extracts. To this end, extracts from *Rlv* UPM791 pea and lentil bacteroids were independently labeled with iTRAQ reagents, mixed, and run through the LC/MS system. Using a conservative threshold value of 2 as significant iTRAQ ratio, we identified 28 proteins overrepresented in pea bacteroids, and eight proteins over-represented in lentil (Table 4). Both methods (iTRAQ and RPSM comparison) gave complementary results, because most of the host-specific proteins detected through RPSM comparison did not allow iTRAQ comparative analysis as they were absent in one of the hosts (supplemental Table S1). In general, proteins showing a pea/lentil iTRAQ value higher than 2 (for pea-overexpressed proteins comparison) or lower than 0.5 (for lentil-overexpressed proteins) also had a RPSM value higher for the corresponding host (Table 4). It has to be noted that both MS-based systems use different detectors, resulting in different peptides detected for the same protein, so a value of 0 RPSM is compatible with iTRAQ values observed in some cases.

**Table 3:** Host-specific proteins identified in pea and lentil bacteroid proteome

Accession	Replicon	Description	RPSM
Pea-specific proteins			
RLV_169	pRlvA	Cytosine deaminase like	21.9
RLV_170	pRlvA	Putative FAD-binding dehydrogenase	37.1
RLV_587	pRlvA	Putative cyclohexadiene dehydrogenase	27
RLV_753	pRlvA	Salicylate hydroxylase	14.3
RLV_1169	pRlvB	Hypothetical protein	3.6
RLV_1358	pRlvB	3-ketoacyl-acyl carrier protein reductase	22.7
RLV_1670	pRlvB	Quinolinate synthetase A	22.7
RLV_1745	pRlvC	Putative ACC deaminase	24.7
RLV_1843A	pRlvC	Cytochrome C biogenesis Redoxin protein	130.8
RLV_1845	pRlvC	ATP-dependent carboxylate-amine ligase	51.7
RLV_1934A	pRlvC	Putative GntR family transcriptional regulator	23.1
RLV_1940	pRlvC	Glutamine--scyllo-inositol aminotransferase	23.5
RLV_1961	pRlvC	Uptake hydrogenase large subunit HupL	43.9
RLV_1962	pRlvC	Uptake hydrogenase small subunit hupS	12.8
RLV_2267	pRlvE	Putative methylmalonyl-CoA mutase	11.8
RLV_2389	pRlvE	Ribitol dehydrogenase	28.1
RLV_3444	Chr	Metal ABC transporter substrate-binding	50.6
RLV_3820	Chr	Molecular chaperone GroEL	24.2
RLV_4312	Chr	Sugar ABC transporter substrate-binding	11.4
RLV_4318	Chr	Oxidoreductase	13.5
RLV_4716	Chr	ABC transporter substrate-binding	10.7
RLV_4843	Chr	FeS assembly SUF system protein	45.8
RLV_4989	Chr	3,4-dihydroxy-2-butanone-4-phosphate synthase	26.7
RLV_5639	Chr	Hypothetical protein	27.2
RLV_5997	Chr	Nitrate ABC transporter substrate-binding	16.7
RLV_6056	Chr	Short-chain dehydrogenase/reductase	36.4
RLV_6236	Chr	Chemotaxis protein	11.9
RLV_6451	Chr	Phytanoyl-CoA dioxygenase	32.2
RLV_7147	Chr	NADH pyrophosphatase	19.4
Lentil-specific proteins			
RLV_95	pRlvA	5-dehydro-4-deoxyglucarate dehydratase	23.6
RLV_96	pRlvA	Putative fatty aldehyde dehydrogenase	16.7
RLV_97	pRlvA	Putative methyltransferase	22.9
RLV_98	pRlvA	Putative mandelate racemase	14.4
RLV_502	pRlvA	Putative small heat shock protein	56.4
RLV_673	pRlvA	Phosphomethylpyrimidine synthase	8.5
RLV_817	pRlvA	Molecular chaperone Hsp20	77.8
RLV_1031	pRlvA	Histidine kinase	4.8
RLV_1519	pRlvB	Hypothetical protein	12.2
RLV_1663	pRlvB	Putative HTH family transcriptional regulator	28.2
RLV_1896	pRlvC	SAM-dependent methyltransferase	69.6
RLV_2599	pRlvE	Putative monooxygenase	34.7
RLV_3334	Chr	RNA-binding	4.8
RLV_4086	Chr	Pyrophosphatase	13.7
RLV_4436	Chr	Protein translocase TatA	100
RLV_4774	Chr	DNA topoisomerase IV subunit B	7.3
RLV_4836	Chr	Shikimate 5-dehydrogenase	27.3
RLV_4908	Chr	Signal transduction histidine kinase	7.6
RLV_5691	Chr	C4-dicarboxylate transporter	31.6
RLV_5754	Chr	Multidrug ABC transporter ATP-binding	6.8
RLV_5939	Chr	Nodulation protein NodT	24.5
RLV_5999	Chr	ABC transporter permease	4.2
RLV_6941	Chr	50S ribosomal protein L27	66.7
RLV_7111	Chr	MerR family transcriptional regulator	24.6
RLV_7210	Chr	Heme ABC transporter ATP-binding	8.9

**Table 4:** iTRAQ comparative analysis of pea and lentil bacteroid proteomes

Accession	Replicon	Description	iTRAQ P/L	RPSM	
				Pea	Lentil
Pea-overexpressed proteins					
RLV_1136	pRlvA	ornithine carbamoyltransferase	2.62	52	25
RLV_1140	pRlvA	Lysine decarboxylase, inducible	2.60	50	14
RLV_1141	pRlvA	Ornithine decarboxylase, inducible	2.20	31	3
RLV_1384	pRlvB	universal stress protein	2.10	167	88
RLV_1399	pRlvB	molecular chaperone Hsp20	2.00	146	89
RLV_1404	pRlvB	phosphoketolase	2.24	39	18
RLV_1805	pRlvC	hypothetical protein	2.45	38	5
RLV_1815A	pRlvC	putative amino-acid racemase	2.06	40	18
RLV_1821	pRlvC	OsmC-like protein	2.23	111	7
RLV_1826	pRlvC	Redoxin	2.29	33	10
RLV_1843A	pRlvC	cytochrome C biogenesis redoxin protein	3.22	131	0
RLV_1844	pRlvC	diaminobutyrate aminotransferase	2.41	182	22
RLV_1845	pRlvC	putative urea amidolyase	2.86	52	0
RLV_1846	pRlvC	D-alanine-- ligase	3.77	111	42
RLV_1848	pRlvC	Glutamyl-tRNA reductase	2.88	128	64
RLV_1849	pRlvC	putative urea amidolyase	2.66	76	35
RLV_1887	pRlvC	putative glutamate dehydrogenase	2.35	81	56
RLV_1889	pRlvC	TlpA like family protein	2.12	44	13
RLV_1961	pRlvC	Uptake hydrogenase large subunit HupL	2.42	44	0
RLV_1979	pRlvC	isocitrate dehydrogenase	2.39	137	102
RLV_4347	Chr	universal stress protein	3.02	129	92
RLV_4576A	Chr	dimethylmenaquinone methyltransferase	2.34	33	10
RLV_4577	Chr	universal stress protein UspA	2.16	90	29
RLV_4675	Chr	nitrogen regulatory protein P-II 1	2.16	70	12
RLV_5449	Chr	GntR family transcriptional regulator	2.66	22	11
RLV_7044	Chr	phosphoenolpyruvate carboxykinase [ATP]	2.24	92	49
RLV_7109	Chr	NAD-dependent succinate-semialdehyde DH	3.24	130	39
RLV_7281	Chr	YciF stress-response, ferritin-like domain containing protein	2.04	33	13
Lentil-overexpressed proteins					
RLV_502	pRlvA	putative small heat shock protein	0.36	0	56
RLV_817	pRlvA	molecular chaperone Hsp20	0.17	0	78
RLV_818	pRlvA	putative small heat shock protein	0.45	18	107
RLV_1833	pRlvC	Transmembr. nitrogen fixation cation transport protein FixI	0.34	1	28
RLV_1892	pRlvC	nitrogen fixation FixC protein	0.42	54	136
RLV_1894	pRlvC	NifA transcriptional regulator	0.39	1	46
RLV_1896	pRlvC	SAM-dependent methyl transferase	0.39	0	70
RLV_5494	Chr	acetolactate synthase	0.50	10	37

iTRAQ, isobaric tags for relative and absolute quantitation.

Proteins identified as pea-specific by both methods included the hydrogenase subunit HupL, an internal control of the system for which we had previous evidence of a strong host-dependent expression (6). The remaining proteins differentially expressed in both hosts belonged to several functional classes including nitrogenase-related proteins, transporters, chaperones/folding catalysts, and transcriptional regulators (Tables 3 and 4).

#### Nitrogenase-Related Proteins

**Fig. 1:**
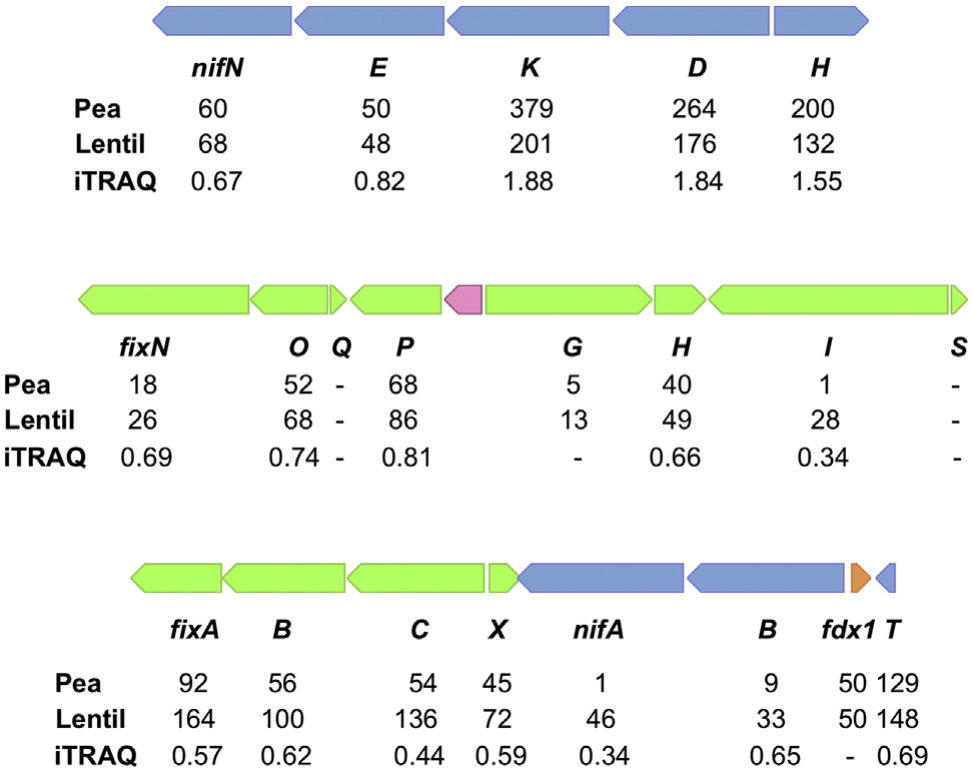
**Proteomic detection of nitrogenase-related proteins in pea and lentil bacteroids**. The numbers below the genetic map of *Rlv* UPM791 nif/fix gene clusters indicate the relative number of assigned spectra (RPSM) in pea and lentil bacteroids and iTRAQ (pea/lentil ratio) values related to the products of the indicated genes. *nif* and *fix* genes are represented by blue and green arrows, respectively. iTRAQ, isobaric tags for relative and absolute quantitation.

Nitrogenase structural proteins, among the most abundant proteins in pea bacteroids, were also quite abundant in lentil bacteroids, although both RPSM and iTRAQ values indicate that there are more abundant in pea than in lentil (supplemental Table S1). Surprisingly, the levels of several accessory proteins involved in the synthesis of the enzyme were higher in lentil, and more interestingly, the level of NifA (RLV_1894), the master regulator of *nif* genes, was clearly more abundant in lentil than in pea nodules, where the level was almost undetectable (Table 4 and supplemental Table S1, and Fig. 1). These data might indicate that the process of nitrogenase synthesis is somehow impaired in the *Rlv* UPM791 symbiosis with lentils as compared with that in pea, thus leading to a relative lower level of nitrogen fixed that the system might tend to compensate by increasing the level of NifA and other proteins involved in nitrogenase synthesis. NifA expression in alfalfa nodules is known to strongly decrease in the mature nodules (17), and a previous report from our laboratory indicate that the specific nitrogenase activity expressed by UPM791 is significantly lower in lentil than in pea nodules (6), suggesting that the symbiosis is not equally efficient in both hosts.

#### Transport Proteins

Five out of the 39 proteins found specifically on pea bacteroids corresponded to PBP from ABC-type transport systems, suggesting that both hosts provide the bacteroids with different nutrient environments.

The annotation of the transport proteins indicate different potential substrates such as metal, nitrate, and sugars, although the actual substrate corresponding to each one remains to be determined. One of the PBP showing the highest number of PSM spectra was RLV_3444, a potential metal-binding component of an ABC transporter system (RLV_3442-43) that is likely more expressed in pea than in lentil. Because both plants were cultured under the same conditions, this result suggests that pea peribacteroid membrane might be more restrictive for providing a specific metal into bacteroids than in the case of lentil. The corresponding genes are highly conserved in *Rlv* 3841 (RL1047 and 1049 being 98 and 99% identical).

Some of the transport proteins identified with a differential expression profile were predicted as integral membrane proteins. One of these proteins (RLV_5691) is the *Rlv* UPM791 ortholog of dicarboxylate permease DctA that was consistently detected in lentil bacteroids but not in pea (supplemental Table S1). It has been shown that the transport of malate through DctA is essential for nitrogen fixation in pea bacteroids (60) so the protein is likely to be expressed in this host, the lack of detection being likely linked to the fact that it is a membrane protein. It has been shown that expression of *dctA* in *R. leguminosarum* is induced under conditions of low nitrogen availability (60), so the higher expression in lentil nodules might be linked to the differences in nitrogenase proteins and activity indicated above.

#### Stress–Response Proteins

Combined analyses based on RPSM and iTRAQ values revealed the existence of several *Rlv* UPM791 stress–response proteins (sHSPs, USPs, and others) whose abundance is host-dependent in bacteroids: three sHSPs (RLV_502, RLV_817, and RLV_818) were overexpressed in lentil bacteroids, and one more member of this family (RLV_1399) was found at higher levels in pea bacteroids. Interestingly, all the sHSPs identified in this way are encoded in plasmids (three in megaplasmid pRlvA and one in pRlvB), thus suggesting that these extrachromosomal DNA incorporate adaptive traits that can be useful for the bacteria to improve survival under conditions found within different hosts. Because the specific targets for these sHSPs are not known, we can only speculate about the molecular basis for this adaptation. This could be related to differences on the profile of unfolded proteins target for sHSP as a consequence of different stressors present in the different hosts. Pull-down experiments with strains overexpressing some of these proteins under controlled conditions (61) are currently underway to elucidate this question.

Another group of host-specific stress-responsive proteins are USPs, three of which (RLV_1384, RLV_4347, and RLV_4577) were overexpressed in pea *versus* lentil bacteroids as deduced from the corresponding iTRAQ values (Table 4). Although the molecular mechanisms through which USP proteins provide stress resistance to cells remain largely unknown (62), these proteins have been associated to protection against oxidative stress and iron deficiency, among other stresses (63). There is no specific information on the role of USP in rhizobia, but interestingly, USP-deficient mutants of different pathogens are compromised in intracellular survival and virulence (64, 65), thus suggesting the possibility of a role of these proteins in adaptation to the endosymbiotic lifestyle.

### A Host-Dependent Transcriptional Regulator Contributes to Optimal Symbiotic Nitrogen Fixation in Pea

Out of ca. 50 GntR-like regulators annotated in the *Rlv* UPM791 genome (35), only two (RLV_1934A and RLV_5449) were consistently detected in bacteroids. Interestingly, the comparative analysis of proteomic profile indicated that both GntR-like proteins were overexpressed in pea. In addition, one MerR-like transcriptional regulator (RLV_7111) was overexpressed in lentil. These regulators might control genes involved in the adaptation of bacteroids to the different hosts. To test this hypothesis, we selected the pea-specific, pSym-located *gntR* gene (RLV_1934A) for a more detailed analysis on the relevance of host-specific proteins. A mutant affected in the corresponding gene was constructed, and wild-type and mutant strains were used as inocula for pea and lentil plants (Table 5). Pea plants inoculated with UPM1418, bearing the mutation in RLV_1934A, showed a statistically significant alteration (28% decrease) of nitrogen accumulation in the shoot as compared with wild type. In contrast, nonsignificant differences in the amount of nitrogen fixed were observed in the case of lentil plants inoculated with the same strain. In both cases, a decrease on shoot dry weight was observed, although the decrease was much more evident in the case on pea plants (28% *versus* 12%). These data indicate a role for RLV_1934A more relevant in pea than in lentil bacteroids. Introduction of a wild-type version of the gene cloned in a plasmid (strain UPM1419) did not revert the phenotype (Table 5). We hypothesize that the presence of the gene in a plasmid might not result in the optimal level of regulator to efficiently complement the symbiotic role of this gene. We are currently investigating the set of genes regulated by this protein to identify the specific factors responsible for this variation in symbiotic efficiency. The involvement of GntR regulators in symbiosis has been described also in *S. meliloti*, whose genome encodes 54 GntR-like regulators. Following systematic mutations of all these genes, it was found that two mutants (affected in SMa0160 and SMa0222, respectively) were associated to impaired symbiosis and reduced competitiveness for nodulation (66).

**Table 5:** Effect of GntR-type transcriptional regulator RLV_1934A on the symbiotic performance of *R. leguminosarum* bv viciae with pea and lentil as host plants

Strain^a^	Pea		Lentil	
	Shoot dry weight (mg/plant)	N fixed (mg/plant)	Shoot dry weight (mg/plant)	N fixed (mg/plant)
Control	162.4 ± 4.3 c	2.22 ± 1.28 c	113.8 ± 10.4 c	1.42 ± 0.11 c
UPM791	482.9 ± 48.5 a	21.96 ± 12.68 a	283.9 ± 14.5 a	7.22 ± 0.42 a
UPM1418	347.8 ± 26.4 b	15.74 ± 9.09 b	235.4 ± 12.2 b	6.39 ± 0.25 ab
UPM1419	327.9 ± 35.4 b	15.31 ± 8.84 b	209.5 ± 26.9 b	5.87 ± 0.69 b
L.S.D.	88.1	3.90	46.0	1.14
CV (%)	17.29	18.35	14.17	14.22

a UPM1418 corresponds to UPM791*gntR*::pK18*mob*; UPM1419 corresponds to UPM1418(pBBGntR). Data are means of four replicates ± standard error. Control: uninoculated and nonfertilized plants. Values followed by the same letter, within each column, are not significantly different at *p* < 0.05.

### Rlv UPM791 Bacteroids Induced in Pea and Lentil Receive Different Sets of NCR Peptides

**Fig. 2:**
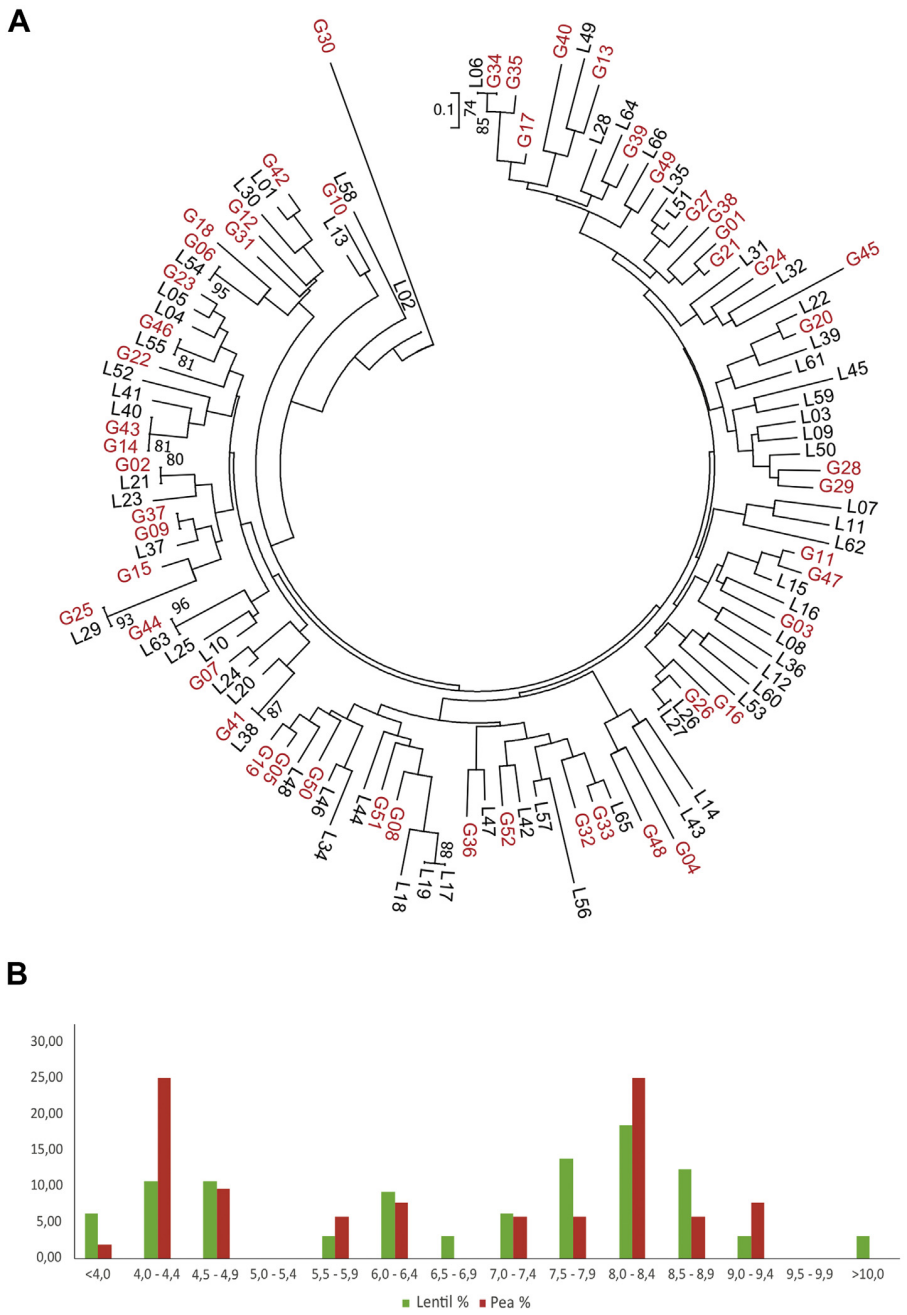
**Analysis of NCR peptides identified in pea and lentil bacteroids**. *A*, phylogenetic relationships of pea and lentil NCR peptides identified in this work. The evolutionary history was inferred using the Neighbor-Joining method. The analysis involved 117 amino acid sequences. Evolutionary analyses were conducted using MEGA7. *B*, distribution of the percentages of anionic (pI 4–6.5), neutral (6.5–7.5), and cationic (7.5–10) NCR peptides identified in bacteroids of *Rlv* UPM791 induced in pea (*red bars*) and lentil (*green bars*). NCR, nodule-specific cysteine-rich.

Pea and lentil are members of the IRLC group of legumes, known to produce NCR peptides that are sent to bacteroids and modify their physiology (14). In the case of pea, the presence of NCR peptides in the nodule has been shown through transcription analysis (14, 67), whereas no previous data of lentil NCR are available. We used the bacteroid proteomic profile to identify potential NCR peptides in the pea and lentil bacteroid protein extracts. To do this, spectra from LC-MS/MS analysis were compared with those generated from the Uniprot Fabaceae database, thus leading to the identification of seven tryptic peptides in the pea bacteroid extracts candidate to belong to NCR peptides described in *P. sativum* (three peptides), *M. truncatula* (three peptides), and *Vicia faba* (one peptide). Only one of these tryptic peptides was found in lentil bacteroids. Because the presence of more NCRs was expected and the proteomes of pea and lentil are not available, a *de novo* search was carried out by further analyzing MS/MS spectra for NCR-characteristic motifs deduced from published NCR sequences (see Supplementary Material). Identified spectra were interpreted manually and with PEAKS software to obtain *de novo* sequences of tryptic fragments that were candidate to be part of NCR peptides. To find the whole sequence of potential NCR peptides whose fragments were identified in the MS/MS analysis, RNA pools obtained from mature nodules of pea and lentil were prepared, selected for poly-A, and sequenced to a depth of 65 Mreads. From this, libraries of ca. 150,000 cDNA contigs from each species were assembled. The sequences were *in silico* translated and used for the identification of additional candidate peptides through BLAST analysis using NCR sequences as queries. Potential NCR peptides identified in the RNA-seq libraries were searched for at the library of unassigned spectra. In this way, a total of 52 and 65 NCR peptides were identified in the proteome of pea and lentil bacteroids, respectively (supplemental Table S2) with virtually not a single sequence fully conserved between the two species. Our data confirm previous findings in similar experiments carried out with *S. meliloti* bacteroids induced in *M. truncatula*, indicating that NCRs are present at high levels in nodule bacteroids and can be consistently detected in complex mixtures (23). Protein sequences of the pea and lentil NCR peptides were aligned, and a phylogenetic tree was deduced from the alignment (Fig. 2*A*). Sequence comparison suggests the existence of parallel evolution in the two hosts but also evolution likely by gene duplication in each host. These data indicate that NCRs constitute a gene family with a high plasticity.

A functionally relevant trait of NCR peptides is their isoelectric point (pI), because cationic peptides will more likely interact with bacterial membranes. Analysis of pIs of the predicted processed forms of NCR identified in this analysis revealed that pIs accumulate around two peaks, with both anionic peptides (pI 4–4.9) and cationic ones (pI 7.5–9) as the main types (Fig. 2*B*). A similar situation was found in other IRLC legumes (14). From the available data, it looks like lentil would produce a higher percentage of cationic peptides (51% *versus* 44% in peas). It has to be noted, however, that we have identified only a fraction of the total of NCRs predicted by transcriptomic analysis.

To confirm the data obtained from proteomics/RNAseq analysis, we searched the preliminary versions of pea (*P. sativum* cv Cameor) and lentil (*L. culinaris* cv Redberry) genomes at the KnowPulse site (https://knowpulse.usask.ca). In this analysis, we found that NCR genes were spread into several chromosomes (supplemental Table S3). Coding sequences were in most cases interrupted by a single intron in the genome. Using high thresholds for identity values (>90%) and cover percentage (>50%), a 91% (48/53) of our pea NCR sequences were identified by using BLASTn in the *P. sativum* genome, with 34 sequences presenting a 100% identity value. In the case of lentil NCR sequences with *L. culinaris* genome, a 92% (60/65) of the sequences presented identity values over 90% and cover percentage over 50%, and from these, 50 sequences had a 100% identity value. Because we had used pea and lentil cultivars different to those sequenced, our results indicate a high level of intraspecific conservation of NCR sequences. However, a few NCRs (two and five sequences in lentil and pea genomes, respectively) gave no hit in the corresponding genome (supplemental Table S3). The different profile of NCR peptides found in lentil *versus* pea bacteroids might be one of the causes leading to the observed host-specific stress responses in *Rlv* UPM791 bacteroids. A similar situation occurs in the *S. meliloti/M. truncatula* system. In this system, *S. meliloti* homologs of two of the small heat-shock proteins over-expressed in lentil bacteroids (RLV_817, and RLV_818) were differentially induced in vegetative cells by two different NCR peptides (68).

The data presented in this work constitute the first proteomic analysis of *R. leguminosarum* bv. viciae bacteroids and show the abundance of nitrogenase proteins, TCA cycle enzymes, and stress–response proteins in endosymbiotic cells. Also, the first sequences of *L. culinaris* NCR peptides are made available and shown to be different, but related, to those produced by *P. sativum*. The results obtained by comparing bacteroids induced in pea and lentil indicate a significant effect of the host in the expression of a set of bacterial proteins that might give clues to further study the adaptation of the bacteria to specific intracellular environments.

## Data availability

The mass spectrometry proteomics data have been deposited to the ProteomeXchange Consortium via the PRIDE partner repository (33) with the dataset identifiers PXD020631 and PXD020634.
