# Supplementary material for: Proteome Analysis Reveals a Significant Host-Specific Response in Rhizobium leguminosarum bv. viciae Endosymbiotic Cells
Source: Mol Cell Proteomics. 2020 Dec 6;20:100009. doi: 10.1074/mcp.RA120.002276 (PMC7950203; doi:10.1074/mcp.RA120.002276)
Supplement: Supplemental Material [file mmc1.pdf]

Proteome analysis reveals a significant host-specific response in *Rhizobium leguminosarum* bv viciae endosymbiotic cells

Durán[#], D., Albareda, M., García, C., Marina, A.I., Ruiz-Argüeso, T., and Palacios, J.M*.

Supplementary material

- Detailed methods for LC-MS/MS analysis and *de novo* sequencing
- Additional references
- List of supplementary Tables
- Supplementary Figures

Detailed Methods for proteome analysis

In solution digestion of cell protein extract

Digestion was carried out essentially as described by Torres et al (25) with some modifications. After denaturation of protein with 8 M urea, the sample was reduced and alkylated: disulfide bonds from cysteinyl residues were reduced with 10 mM DTT for 1 h at 37 ºC, and then thiol groups were alkylated with 50 mM iodoacetamide for 1 h at room temperature in darkness. The sample was diluted to reduce urea concentration below 1.4 M and digested using sequencing grade trypsin (Promega, Madison, WI) overnight at 37 ºC using a 1:20 (w/w) trypsin/protein ratio. Digestion was stopped by the addition of 1% TFA. Whole supernatants were dried down and then desalted onto OMIX Pipette tips C18 (Agilent Technologies) until the mass spectrometric analysis.

Reverse phase-liquid chromatography RP-LC-MS/MS analysis

For LC chromatography and MS/MS analysis we follow essentially the procedure described by Alonso et al (68), with some modifications. The desalted protein digest was dried, resuspended in 10 µl of 0.1% formic acid 4% acetonitrile and analyzed by RP-LC-MS/MS in an Easy-nLC II system coupled to an ion trap LTQ-Orbitrap-Velos-Pro hybrid mass spectrometer (Thermo Scientific). The peptides were concentrated (on-line) by reverse phase chromatography using a 0.1mm × 20 mm C18 RP precolumn (Thermo Scientific), and then separated using a 0.075mm x 250 mm C18 RP column (Thermo Scientific) operating at 0.3 µl/min. Peptides were eluted using a 180-min dual gradient from 5 to 25% solvent B in 135 min followed by gradient from 25 to 40% solvent B over 180 min (Solvent A: 0,1% formic acid in water, solvent B: 0,1% formic acid, 80% acetonitrile in water). ESI ionization was done using a Nano-bore emitters Stainless Steel ID 30 µm (Proxeon) interface. The system was set at a resolution ($m/\Delta m$) of 30,000. Peptides were detected in survey scans from 400 to 1600 amu (1 µscan), followed by twenty data dependent MS/MS scans (Top 20), using an isolation width of 2 u (in mass-to-charge ratio units), normalized collision energy of 35%, and dynamic exclusion applied during 30 seconds periods.

iTRAQ labelling

The peptide mixture resulting from protein tryptic digest (100 µg) was labeled using chemicals from the iTRAQ reagent 8plex Multi-plex kit (reagents 113, 114, 115, 116, 117, 118, 119 and 121) (Applied Biosystems) essentially as described (26). Briefly, peptides were dissolved in 0.5 M triethylammonium bicarbonate (TEAB), adjusted to pH 8. For labeling, each iTRAQ reagent was dissolved in 50 µL of isopropanol and added to the respective peptide mixture and then incubated at room temperature for two hours. Labelling was stopped by the addition of 0.1% formic acid. Whole supernatants were dried down and the eight samples were mixed to obtain the "8plex-labeled mixture". The mixture was desalted onto OASIS HLB Extraction Cartridges (Waters Corporation) until the mass spectrometric analysis.

RP-LC MS/MS analysis of iTRAQ-labelled samples

The desalted 8plex-labeled mixture was dried, resuspended in 10 µl of 0.1% formic acid and analyzed by RP-LC-MS/MS in an Easy-nLC II system coupled to an ion trap LTQ-Orbitrap-Velos-Pro hybrid mass spectrometer (Thermo Scientific). The peptides were concentrated (on-line) by reverse phase chromatography using a 0.1 mm × 20 mm C18

RP precolumn (Proxeon), and then separated using a 0.075 mm x 250 mm C18 RP column (Proxeon) operating at 0.3 µl/min. Peptides were eluted using a 240-min dual gradient from 5 to 25% solvent B in 180 min followed by gradient from 25 to 40% solvent B over 240 min (Solvent A: 0,1% formic acid in water, solvent B: 0,1% formic acid, 80% acetonitrile in water). ESI ionization was done using a Nano-bore emitters Stainless Steel ID 30 µm (Proxeon) interface (Alonso et al., 2015).
The instrument method consisted of a data-dependent top-20 experiment with an Orbitrap MS1 scan at a resolution ($m/\Delta m$) of 30,000 followed by either twenty high energy collision dissociation (HCD) MS/MS mass-analyzed in the Orbitrap at 7,500 ($\Delta m/m$) resolution. MS2 experiments were performed using HCD to generate high resolution and high mass accuracy MS2 spectra. The minimum MS signal for triggering MS/MS was set to 500. The lock mass option was enabled for both MS and MS/MS mode and the polydimethylcyclosiloxane ions (protonated (Si(CH3)2O))6; *m/z* 445.120025) were used for internal recalibration of the mass spectra.
Peptides were detected in survey scans from 400 to 1600 amu (1 µscan) using an isolation width of 2 u (in mass-to-charge ratio units), normalized collision energy of 40% for HCD fragmentation, and dynamic exclusion applied during 30 seconds periods. Precursors of unknown or +1 charge state were rejected.

Data Analysis
Peptide identification from raw data was carried out using the SEQUEST algorithm (Proteome Discoverer 1.4, Thermo Scientific). Database search was performed against Uniprot-Fabaceae.fasta, the proteome deduced from *Rlv* UPM791 genome (Sanchez-Cañizares et al., 2018), and in-silico translation of RNA-seq contigs from pea and lentil bacteroids. The following constraints were used for the searches: tryptic cleavage after Arg and Lys, up to two missed cleavage sites, and tolerances of 20 ppm for precursor ions and 0.8 Da for MS/MS fragment ions and the searches were performed allowing optional Met oxidation, Cys carbamidomethylation, and iTRAQ reagent labeling at the N-terminus and lysine residues. Search against decoy database (integrated decoy approach) using false discovery rate (FDR) < 0.01.
Quantitation of iTRAQ labeled peptides was performed with Proteome Discoverer 1.4 using a Workflow for processing raw files with HCD spectra for quantification (and identification). The Reporter Ions Quantifier node contains a specific quantification method for i-TRAQ 8plex (Thermo Scientific Instruments). For the ratio calculations we use Quan Value corrections and for the Protein quantification we consider protein groups for peptide uniqueness and use only unique peptides. Tolerances of 10 ppm for peak integration and most confident centroid for integration method were selected.

De Novo Sequencing for NCR identification
MS/MS spectra with a good quality that were not confidently assigned to a database peptide/protein were interpreted manually and with PEAKS 8 software to obtain *De Novo* sequences following the workflow summarized in Fig. S2. The sequences were used for the identification of NCR peptides based on homologies using BLAST searches on database generated from *in*-silico translated RNA-seq derived contigs.

Supplementary Tables

Table S1. Proteomic detection (RPSM and iTRAQ) of proteins in *Rlv* UPM791 pea and lentil bacteroids, and in vegetative cells.

Table S2. Amino acid sequences of NCR peptides identified in pea and lentil bacteroids. Only the processed forms of the peptides are shown.

Table S3. Localization of genes encoding NCR peptides identified in bacteroid proteomes in the draft genomes of pea (*P. sativum* cv Cameor) and lentil (*L. culinaris* cv. Redberry).

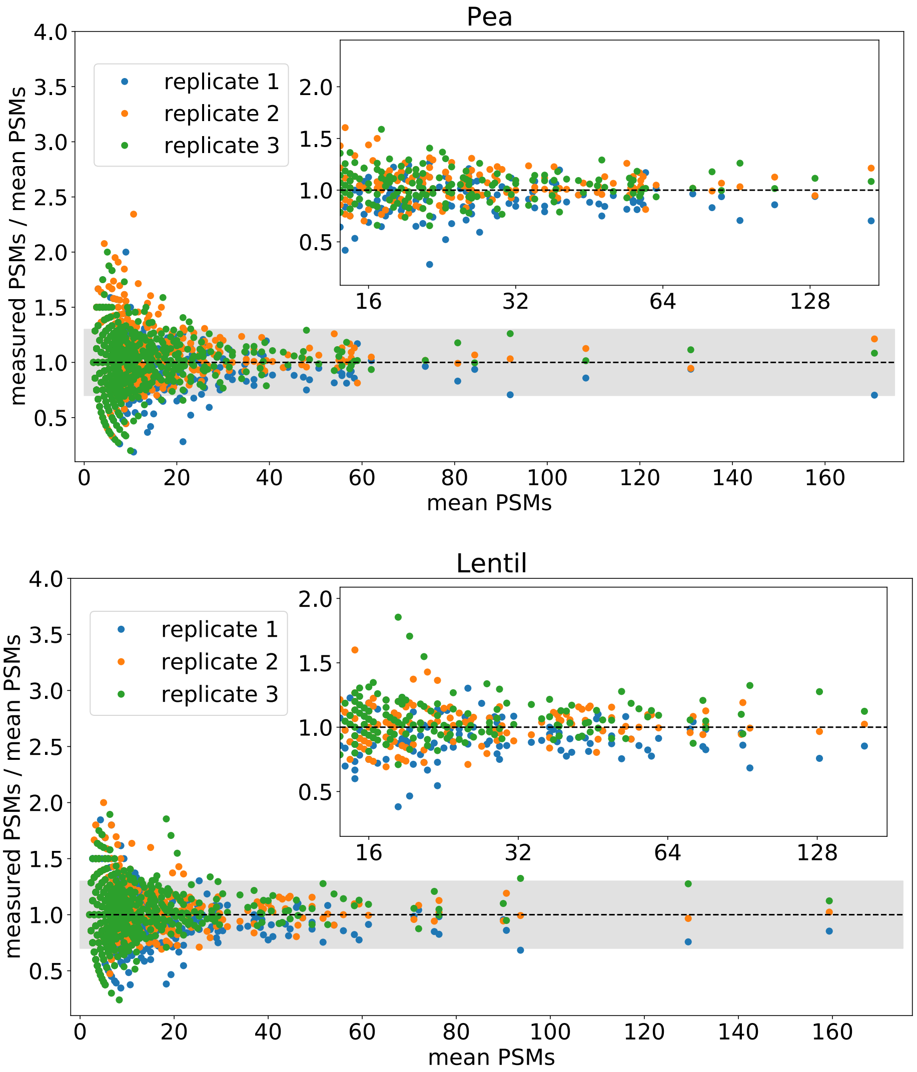

Figure S1. Dispersion of PSM values in replicates from pea (top) and lentil (bottom) bacteroid proteomic determinations. Dots of different colors correspond to PSM values in replicates as regarding mean values. Data are ordered according to the value of the mean in Table S1. Only proteins detected in all three replicates are represented. The area representing dispersion values ≤ 30% is shadowed. Figure in inset includes a log2 transformation of x-axis for clarity.

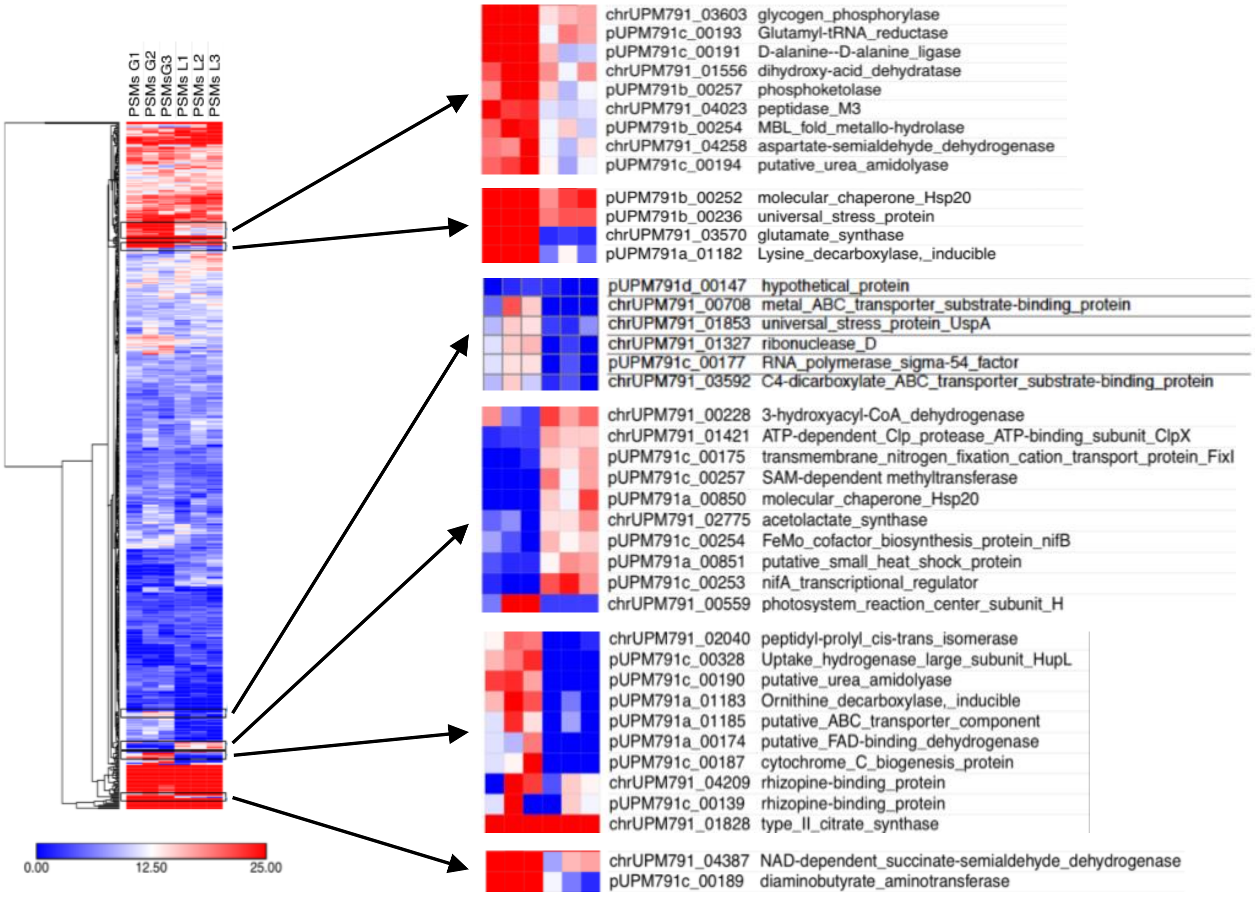

Figure S2. Heatmap from comparative proteomic analysis of bacteroids induced by *Rlv* UPM791 in pea and lentil nodules. Lanes correspond to biological replicates of bacteroids from pea (G1-G3) and lentil (L1-L3). Protein designation refers to locus accessions in Table S1. Heatmap was generated using Morpheus software available at https://software.broadinstitute.org/morpheus. Values correspond to the number of PSM detected for each protein.

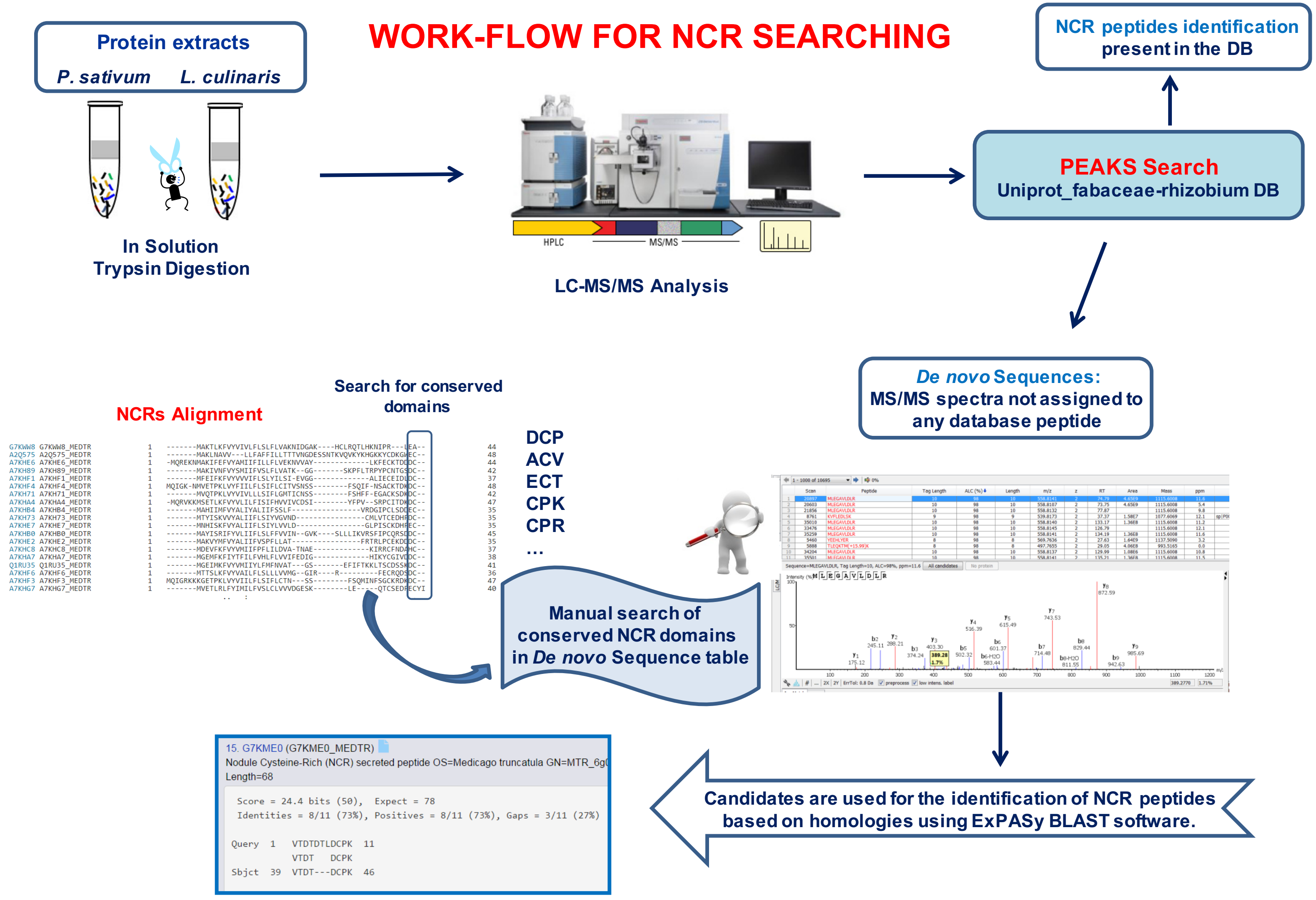

Figure S3. Workflow for *de novo* identification of NCR peptides in unassigned spectra
A PEAKS search in the compiled database (Fabaceae-rhizobium) to identify all the legume and rhizobium proteins, as well as the NCR peptides that are included in the database. The program generates a list of spectra called *de novo only* which includes all MS / MS spectra that do not correspond to any compiled database peptides.
This *de novo* list was used to identify NCR peptides. First we generate an alignment of all the NCR sequences present in UniProt DB and look for all the conserved sites. With tags of three amino acids we manually search in the *de novo* MS/MS spectra. Candidates were used for the identification of NCR peptides based on homologies using ExPASy BLAST software.
